# Prevalence of Colistin-Resistant *Escherichia coli* from Poultry in South Asian Developing Countries

**DOI:** 10.1155/2021/6398838

**Published:** 2021-10-11

**Authors:** Prabin Dawadi, Shrijana Bista, Sayara Bista

**Affiliations:** ^1^Nepal Academy of Science and Technology, Khumaltar, Lalitpur, Nepal; ^2^Central Department of Microbiology, Tribhuvan University, Kirtipur, Kathmandu, Nepal

## Abstract

**Background:**

Antimicrobial resistance has become a huge problem in animal and public health worldwide. Inadvertent use of antibiotics in poultry has led to the emergence of resistance against many antibiotics, even to last resort of drugs like colistin.

**Aim:**

This study aimed to provide uniform quantitative estimate on the percentage prevalence of *E*. *coli* as well as to analyze their colistin resistance in poultry in South Asian developing countries.

**Methods:**

Two electronic databases, PubMed and Research4Life, were used to search studies as per the Preferred Reporting Items for Systematic Reviews and Meta-Analysis (PRISMA) guidelines. The pooled data on the prevalence of *E*. *coli* and colistin resistance were analyzed.

**Results:**

In the meta-analysis of 9 studies in South Asian region (Nepal, Bangladesh, Pakistan, and India), the pooled prevalence of *E*. *coli* was 73% (95% CI, 0.549–0.916). The pooled prevalence of colistin resistance against *E*. *coli* from poultry was found to be 28% (95% CI, 0.158–0.438).

**Conclusion:**

There is high prevalence of *E*. *coli* and their resistance rate against colistin in poultry. Therefore, antimicrobials in raising livestock should be supervised.

## 1. Introduction

*Escherichia coli* are normal intestinal microflora in birds [1]. Avian pathogenic *E*. *coli* (APEC) invades different organs of birds causing localized or systemic infections termed as Extraintestinal pathogenic *E*. *coli* [2]. It is characterized by pericarditis, air sacculitis, perihepatitis, peritonitis and other extraintestinal diseases, referred as colibacillosis [3, 4].

The use of antibiotics in compound feeds has been an integral part of poultry production, not only to prevent infectious bacterial diseases but also to promote growth of livestock. Almost all poultry feed industries commonly use different types of antibiotics as feed additives in compound feed, pelleted, or mash, with the objective to enhance growth and feed efficiency. This continuous use of several types of antibiotic molecules at the subtherapeutic level in feeds promotes the bacterial resistance in poultry [5]. Commonly used antibiotic in poultry for the treatment of colibacillosis and for growth promoter of poultry is colistin [6]. Antimicrobial resistance has become a worldwide problem in animal and public health. Inadvertent uses of antibiotics in poultry have led to the emergence of multidrug-resistant organisms causing possible threat to human health [6, 7]. In developing countries like Nepal, poultry farming has become a major source of income to the farmers due to high demand of poultry meat [8, 9]. Antibiotic-resistant *E*. *coli* are the major cause of morbidity and mortality in poultry that leads to high economic loss of the country [10]. Drug-resistant *E*. *coli* are harbored in different organs of poultry that can be easily transferred to humans through food chain or direct contact. The drug-resistant *E*. *coli* acts as a reservoir of drug-resistant genes that can be transferred to human pathogens [11, 12]. It is predictable that inadvertent use of colistin in poultry has contributed to spread of colistin resistance [13]. Discovery of plasmid transferred mobilized colistin resistance (*mcr-1*) gene in animal food in *E*. *coli* lead to worldwide concern of horizontal transfer of resistant genes in human pathogens as well [14].

In order to provide a reliable, uniform quantitative estimate on the percentage prevalence of *E*. *coli* and colistin resistance in poultry in South Asian regions, we used a meta-analytic approach by using data accumulated from published literature after descriptive analyses of the same for some key variables. The obtained results deliver a better estimate of the prevalence of *E*. *coli* and colistin resistance in poultry that could cause horizontal transfer of colistin-resistant genes in humans.

## 2. Methods

This review was conducted using Medline/PubMed, and Research4Life by following the PRISMA guideline [15] (Supplementary file 1 for publication bias). The following search terms were used: “*E*. *coli* and poultry” and “*E*. *coli* resistance poultry South Asia.” The searches were limited by articles published from 2014 to 2021 with work duration ranging from January 2014, until March 2021 in English language.

### 2.1. Eligibility Criteria

Eligibility of studies was determined separately after reviewing search results, and any discrepancies were resolved through discussion among all authors. Any disagreements that arose during the review of full papers were resolved by a majority vote. The initial search strategy's findings were screened by title and abstract. For inclusion and exclusion criteria, the full texts of relevant papers were reviewed.

### 2.2. Inclusion Criteria

Observational studies that reported the prevalence of *E*. *coli* in poultry in South Asian regions along with AST (antibiotic susceptibility test) were included for quantitative synthesis.

Study design type prospective/retrospective cohort study:

1. Articles published in English language.
2. Article language limit was set to English, and publications from 2014 to 2021 with work duration ranging from January 2014 until March 2021 were included.

### 2.3. Exclusion Criteria

Case reports were not included for the systematic analysis, as they do not have a denominator for any variables, but descriptive statistics were applied to them, to summarize our findings.

Studies documenting cases with missing information, as well as review papers, opinion articles, and letters that did not provide original data, were removed from the analysis.

## 3. Methods of Data Extraction

From the screened articles, items including first author, type of the study, the publishing institution, date of publication, site of study, sample size, altitude, and antibiotic resistance with various antibiotics were included (Table 1 in Supplementary Materials).

Article language limit was set to English, and publications from 2014 to 2021 with work duration ranging from January 2014 until March 2021 were included.

Review articles, opinion articles, and letters not presenting original data as well as studies reporting cases with incomplete information were excluded from the study.

### 3.1. Data Synthesis and Analysis

From the screened articles, items including first author, type of study, the publishing institution, date of publication, site of study, sample size, altitude, and colistin resistance were included (Table 1 in Supplementary Materials). All statistical analyses were performed using R language (meta package). Percentages were calculated to describe the distributions of categorical variables. The prevalence of *E*. *coli* infection in poultry was expressed as proportion and 95% confidence interval using the random effects model and was presented as forest plot. Cochran *Q* test was used to detect heterogeneity among studies, with a *p* value < 0.10 indicating significant heterogeneity. *I*^2^ statistic was calculated to measure the proportion of total variation in study estimates attributed to heterogeneity. *I*^2^ values of <25%, 25–75%, and >75% indicate low, moderate, and high heterogeneity [16].

## 4. Results

### 4.1. Summary of the Selected Study

**Figure 1:**
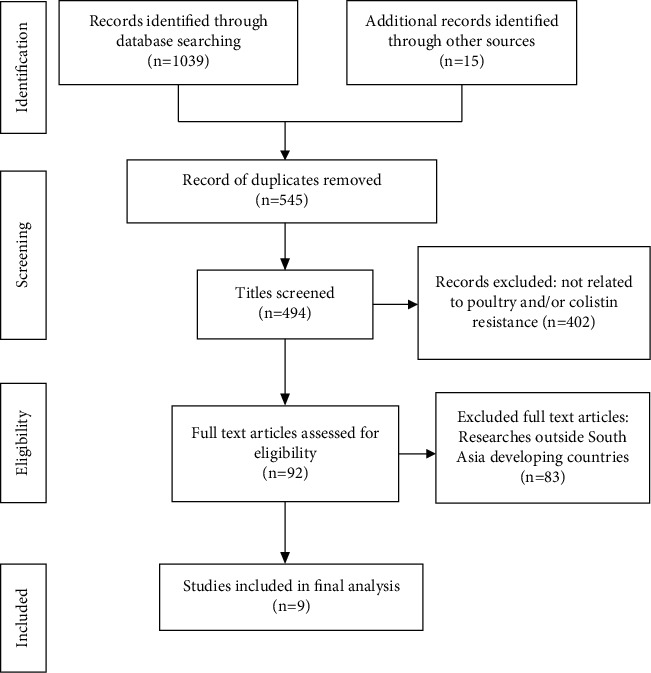
A flow diagram of the search strategy according to the Preferred Reporting Items for Systematic Reviews and Meta-Analyses (PRISMA) guidelines.

Using the search strategy, a total of 1039 potentially important articles were found. After eliminating 545 duplicates, the remaining 494 papers were screened further by title and abstract, with 92 being chosen for full-text evaluation. Nine papers were included in the quantitative meta-analysis, while 83 articles were excluded due to a lack of complete information on antibiotic resistance and studies being conducted beyond South Asian region. The flowchart of study selection is shown in Figure 1.

### 4.2. Meta-Analysis Results

#### 4.2.1. Meta-Analysis on the Prevalence of *E*. *coli*

**Figure 2:**
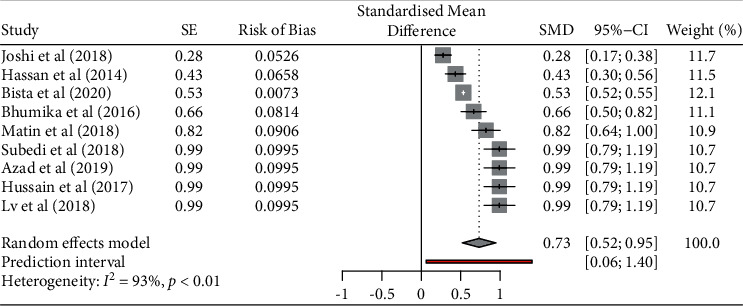
Prevalence of *Escherichia coli* from poultry.

The pooled prevalence of *E*. *coli* from poultry in nine studies was 83.5% (95% CI, 0.651–0.932), with significant heterogeneity noted among studies (*p* < 0.001; *I*^2^ = 92.740) (Figure 2).

### 4.3. Colistin resistance

**Figure 3:**
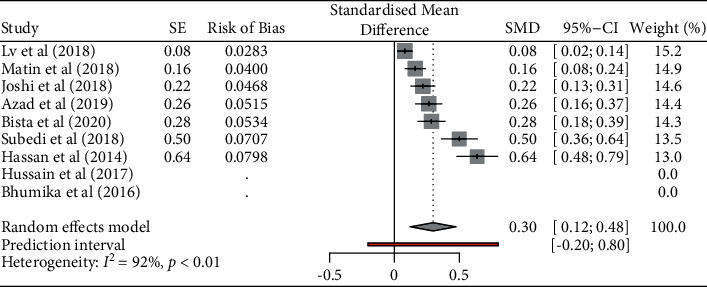
Prevalence of colistin resistance against *Escherichia coli*.

The pooled prevalence of colistin resistance against *E*. *coli* was 30% (95% CI, 0.158–0.438). There was significant heterogeneity among analyzed studies (*p* < 0.001, *I*^2^ = 93.639) (Figure 3).

## 5. Discussion

Antibiotics have been used extensively and haphazardly in poultry as a growth promoter or for treatment purpose in feed, water, and vaccines. This has become a major predisposing factor for bioaccumulation of antibiotics in different organs of poultry. *E*. *coli* have become resistant to last resort of drug, colistin, due to continuous exposure. Humans being continuously exposed to poultry in one or the other way have high chances of acquiring antimicrobial resistance to *E*. *coli*. In such infections, chromosomal or plasmid encoded genes might be transferred among the pathogens and cause possible threat to antimicrobial resistance. Since antimicrobial resistance (AMR) is an emerging global health problem, there is a need to assess its potential clinical and public health impact [9].

The prevalence of *E*. *coli* and antimicrobial resistance pattern determined from poultry samples from 2011 to 2019 was assessed in this meta-analysis. To the best of our knowledge, this is the first meta-analysis study on the prevalence of antimicrobial resistance in poultry from SAARC countries. Providing the vision of antimicrobial resistant patterns of the *E*. *coli* from poultry in SAARC countries may help prevent the spread of antimicrobial resistance in South Asian countries where the study of antimicrobial resistance is lacking compared to other countries of the world. Furthermore, the result generated so far would help to provide background of antibiotic-resistant *E*. *coli* to prevent dissemination of pan drug-resistant pathogen from animals to humans [9]. Narrow searches in the study led to the reduced inclusion of studies in meta-analysis. Hence, 9 studies were taken for meta-analysis.

Overall, the review revealed the prevalence of *E*. *coli* in poultry is very high. *E*. *coli* being a normal inhabitant of the gastrointestinal tract is a normal intestinal microflora of bird and can be present in cloacal swab, caecum, and faeces [1]. However, certain strains of *E*. *coli* can cause a common disease in poultry known as colibacillosis where it invades different organs of birds such as liver, kidney, and spleen [3]. Hence, *E*. *coli* has been isolated from both healthy and infected population.

Based on meta-analysis of the study, pooled prevalence of *E*. *coli* from poultry in 9 studies conducted from South Asian developing countries was 73% (95% CI, 0.549–0.916) with significant heterogeneity (*p* < 0.001; *I*^2^ = 95.66). The prevalence is more compared to other countries. A study in China showed the prevalence of *E*. *coli* to be 4.3% (95% CI, 3.3–5.2) [17]. Differences in prevalence may be due to the level of hygiene practiced in different geographical locations and environmental conditions which includes exposure to decreased sanitation that leads to high prevalence of *E*. *coli* that spreads into various internal organs and cause systematic fatal disease in poultry. Humans acquire these infections through direct contact with poultry in the farm and slaughter-house or due to improperly cooked meat and eggs. Poultry health management along with biosecurity measures are the emerging issues which can be a major cause of zoonotic disease transmission in human. Besides geographical locations and environment, the number of birds in a flock also plays a major role in isolation of high number of *E*. *coli*. Studies show high prevalence of *E*. *coli* has been obtained from larger flocks compared to smaller flocks [4]. This may be due to exposure to decreased sanitation of environment including faecal contamination of feeds and eggs and delay in collection of dead birds.

Minimum inhibitory concentration method was used for determining the resistivity and sensitivity pattern of the isolates in colistin susceptibility assay. Uncontrolled use of antibiotics as well as dietary supplement could be the major cause of antimicrobial resistance. High colistin resistance among *E*. *coli* isolates has been reported (pooled prevalence, 30%) from this study compared to other studies conducted in Europe. In spite of frequent application of colistin in animal farming in Europe, colistin resistance was reported to be less than 1% in animal food. However, *E*. *coli* isolates obtained from poultry in 2007-2008 in Europe showed low frequency of colistin resistance (12.4%) compared to isolates from 2013 to 2014 [18]. Elevated level of colistin to the isolates in this study is probably due to the continuous exposure of E. *coli* to colistin because of its use in feed additives for boosting the growth and performance of poultry or for therapeutic purpose. The exponential decrease in the prevalence of colistin resistance in other countries may be due to the decrease in use of colistin because in 2008, the Ministry of Agriculture, Livestock, and Supply (MAPA) established appropriate levels for colistin use in broilers (2 to 10 g/ton of feed), poultry (4 to 10 g/ton of feed), pigs (20 to 40 g/ton of feed), and cattle (5 to 40 g/ton of feed).

The condition is worrisome since these resistant bacteria might be transferred to humans by food chain as poultry meat is largely consumed by humans or to workers via direct animal contact. Further coordinated action for control and prevention should be started from veterinary level. Antimicrobials in raising livestock should be supervised, and their inadvertent use should be limited. Bacteriology confirmation is required before prescribing any antibiotics [19].

However, after the presence of colistin-resistant *E*. *coli* carrying the mcr-1 gene was confirmed in humans and animals (including livestock), the use of colistin in animal feed was banned by MAPA in November 2016, following the international recommendations of the World Health Organization. India has also banned the use of human critical antibiotic in poultry farm in 2019 [20]. In Bangladesh, the Animal Feed Act prohibits the use of antibiotics in feed [21]. However, poultry farmers outwit the law by including antibiotics in drinking water provided to broilers. In Nepal, the Ministry of Livestock Development (MoLD) has promoted a policy of zero antibiotics in feed supplements, so as to prohibit the use of antimicrobials as growth promoters and to implement strict awareness program to stop the use of antimicrobials at subtherapeutic doses [22].

The limitation of the current study was the lack of comprehensive study from different regions. All the countries of the South Asian developing countries could not be included in the study due to unavailability of the reports. More study on prevalence of organisms along with the antibiotic resistivity patterns from poultry is needed. Such studies could give a broader prospective of AMR pattern of organisms in poultry and help to limit the resistant strains that have high chances of infective humans as well.

## 6. Conclusion and Recommendation

In the South Asian developing countries, the prevalence of *E*. *coli* from poultry is indicated to be high. Resistance patterns in poultry are on the rise as the colistin resistance has reached up to 30%. This suggests further in-depth research into the process and reasons for the colistin resistance in *E*. *coli* with search of alternative antibiotic. Therefore, it is recommended that antimicrobials in livestock production should be closely monitored.

## Data Availability

The data used to support the findings of this study are available from the corresponding author upon request.

## Supplementary Materials

Supplementary Materials Table 1: location and prevalence of isolated *E. coli* and colistin resistance from poultry.
