# Supplementary material for: Prevalence of Colistin-Resistant Escherichia coli from Poultry in South Asian Developing Countries
Source: Vet Med Int. 2021 Oct 11;2021:6398838. doi: 10.1155/2021/6398838 (PMC8523263; doi:10.1155/2021/6398838)
Supplement: Supplementary Materials — Table 1: location and prevalence of isolated E. coli and colistin resistance from poultry. [file 6398838.f1.zip › 6398838.f1/Supplement file1 (1).docx]

| **Author** | **Country** | **Location** | **Duration** | **Type of sample** | **Prevalence** | **Colistin resistance** |
| --- | --- | --- | --- | --- | --- | --- |
| Subedi et al (2018) [25] | Nepal | Chitwan | May 2016-March 2017 | Liver (Colibacillosis) | 100% | 50.00% |
| Joshi et al (2018) [9] | Nepal | Valley, Kaski | Nov 2017-July 2018 | Meat | 27.65% | 21.90% |
| Matin et al (2017) [26] | Bangladesh | Mymensingh | Jul-Dec 2013 | Liver, Spleen, Faeces (Inf) | 82% |  |
| Hassan et al (2014) [28] | Bangladesh | Chittagong | Sep - Dec 2012 | Liver, Spleen | 43.33% | 63.75% |
| Azad et al (2019) [29] | Bangladesh | Rajshahi, Dhaka |  | Cloacal swab | 100% | 26.50% |
| Hussain et al (2017) [30] | India | Tamil nadu | Feb- Sep 2015 | Meat/Ceca | 100% |  |
| Bhumika et al (2016) [31] | India | Chattisgarh | Aug 2014-July 2015 | Meat | 66.32% |  |
| Lv et al (2018) [32] | Pakistan | Faisalabad | Dec 2016- Jan 2017 | Cloacal swab | 100% | 8% |
| Bista et al (2020)[30] | Nepal | Kathmandu | Febraury 2019- July 2019 | Liver samples | 53.3% | 28.5% |

**Prevalence of Colistin Resistant *Escherichia coli* from Poultry in South Asian Developing Countries**

Prabin Dawadi^2^, Shrijana Bista^1^, Sayara Bista*^2^

^1^Central Department of Microbiology, Tribhuvan University, Kirtipur, Kathmandu, Nepal

^2^Nepal Academy of Science and Technology, Khumatar, Lalitpur, Nepal

Correspondence: Sayara Bista, Nepal Academy of Science and Technology, Khumaltar, Lalitpur, Nepal. Email: saybista@gmail.com

**Table 1:** Location and prevalence of isolated *E. coli* and colistin resistance from poultry
