# Supplementary figures and images for: Prevalence of Colistin-Resistant Escherichia coli from Poultry in South Asian Developing Countries
Source: Vet Med Int. 2021 Oct 11;2021:6398838. doi: 10.1155/2021/6398838 (PMC8523263; doi:10.1155/2021/6398838)

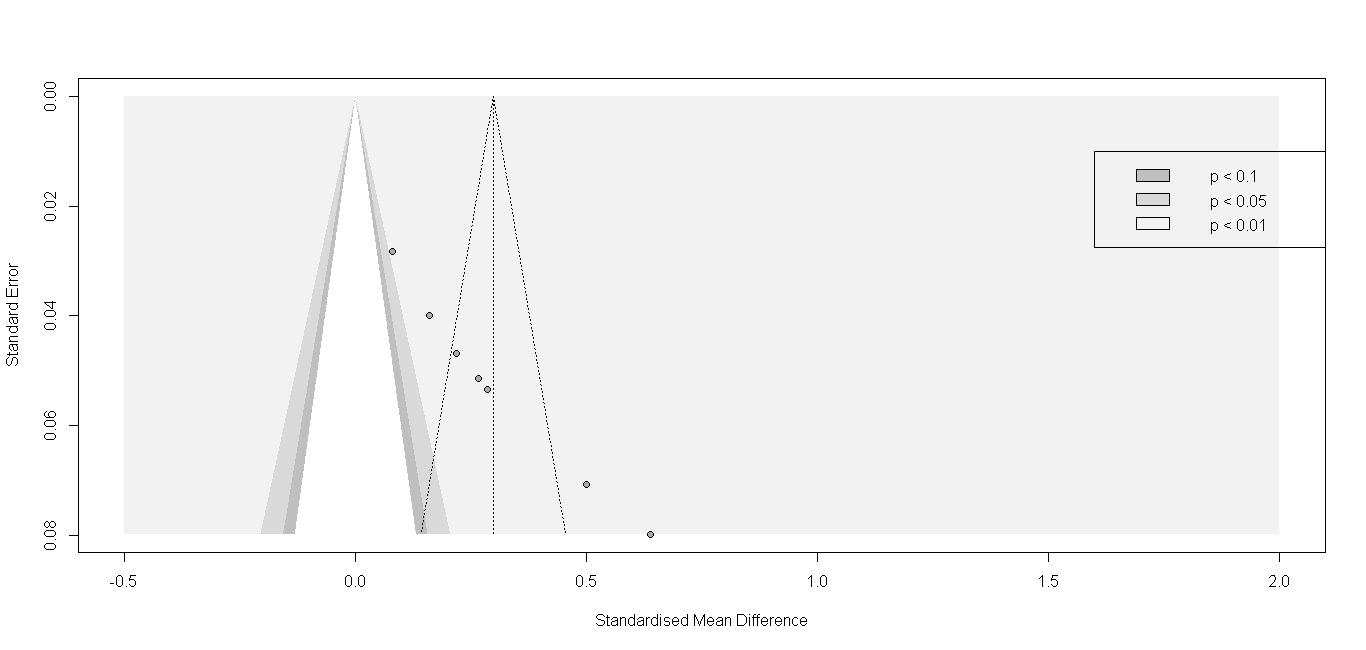

Supplement: Supplementary Materials — Table 1: location and prevalence of isolated E. coli and colistin resistance from poultry. [file 6398838.f1.zip › 6398838.f1/Publication bias.jpeg]
